# Fecal or bacterial transplantation in mice transfer environment-induced brain plasticity and associated behavioral changes

**DOI:** 10.3389/fphys.2025.1572854

**Published:** 2025-05-30

**Authors:** Francesco Marrocco, Rizwan Khan, Alice Reccagni, Xingzi Lin, Mary Delli Carpini, Valerio Iebba, Giuseppina D’Alessandro, Cristina Limatola

**Affiliations:** ^1^ Department of Physiology and Pharmacology, Sapienza University, Rome, Italy; ^2^ Gustave Roussy Cancer Campus, ClinicObiome, Villejuif, France; ^3^ IRCCS Neuromed, Pozzilli, Italy; ^4^ Laboratory affiliated with Instituto Pasteur, Department of Physiology and Pharmacology, Sapienza University, Rome, Italy

**Keywords:** enriched environment, fecal material transplantation, animal behavior, neurogenesis, gut-brain axis

## Abstract

**Introduction:**

Recent studies have shown that lifestyle factors, including diet and environmental stimuli, significantly alter the composition of gut microbiota and the metabolites they produce. Specifically, housing mice in an enriched environment (EE) enhances the production of short-chain fatty acids, which in part mediate the effects of EE on brain plasticity. In this study, we tested the hypothesis that the gut microbial composition of EE-exposed mice could be transplanted into mice housed in a standard environment (SE) to replicate the environmental effects on behavior, gene expression and neurogenesis.

**Methods:**

To test this hypothesis, we transplanted either a specific bacterial mixture or fecal material from EE-housed mice into SE-housed mice.

**Results:**

Our data show that both bacterial and fecal transplants reduce anxiety-like behaviors in mice. Additionally, we observed increased expression of hippocampal neurotrophins and enhanced neurogenesis.

**Discussion:**

These findings support the idea that gut microbiota influence brain functions, including anxiety-like behavior. Further research is necessary to clarify the underlying mechanisms. Moreover, the results suggest that fecal material transplantation (FMT) from individuals with healthy lifestyles may represent a promising strategy for the treatment of mood disorders.

## 1 Introduction

The gut microbiota consists of the microbial community residing in the gastrointestinal tract. These microorganisms are crucial for various biochemical processes, including the digestion of diet-derived fiber (Rosenberg and Zilber-Rosenberg, 2016), which leads to the production of short-chain fatty acids (SCFAs). SCFAs, in turn, influence the growth of other microbes and play a role in modulating both local and peripheral immune functions (Han et al., 2017; Jandhyala, 2015). The composition of the gut microbiota is not static and undergoes changes from birth, adapting to different stimuli such as diet (Palmer et al., 2007), medications, and physical exercise (Sommer and Bäckhed, 2013). Despite these fluctuations, the adult microbiota remains relatively stable, with approximately 90% of the bacterial population belonging to the Firmicutes and Bacteroidetes phyla (Costello et al., 2009). The dynamic balance between the host and its commensal microbes provides several benefits, including maintaining gut barrier integrity, aiding in food digestion, and storing energy (Rowland et al., 2018). Disruptions to this balance can lead to adverse effects on host homeostasis, contributing to metabolic disorders such as diabetes and obesity (Al Bataineh et al., 2023; Contreras-Rodriguez et al., 2022), as well as neurodevelopmental and psychiatric conditions like autism spectrum disorder, anxiety, and depression (Felice and O’Mahony, 2017; Xu et al., 2021). Recently, fecal material transplantation (FMT) has emerged as a potential therapeutic approach for gut dysbiosis, using fecal material from healthy donors, with promising results observed in both animal models and human trials (Xu et al., 2021). However, the mechanisms underlying these beneficial effects remain poorly understood. Recent studies have explored the impact of housing mice in an enriched environment (EE) on gut microbiota and metabolome composition (Lupori et al., 2022; Marrocco et al., 2022). EE, characterized by enhanced motor, sensory, and social stimuli, has been shown to boost hippocampal neurogenesis, improve learning and memory, and reduce depressive-like behaviors (Chen et al., 2024a). On a molecular level, these changes are linked to increased expression of neurotrophins such as BDNF (Cancedda et al., 2004) and NGF (Birch et al., 2013). Additionally, EE has been associated with higher levels of fecal SCFAs, particularly formate and acetate, and distinct microbial populations compared to mice housed in standard environments (SE). These SCFAs are thought to mediate some of the beneficial effects of EE on the brain (Lupori et al., 2022; Marrocco et al., 2022). In this study, we investigated whether the bacterial strains associated with EE could transfer an “enriched” phenotype to control mice. We also performed FMT from EE-housed mice to control mice to evaluate the effects of the complete gut material—microbes and metabolites—on brain function. Our findings show that both bacterial transplantation and FMT alleviated anxiety-like behavior and increased BDNF expression in the hippocampus. Notably, FMT had more pronounced effects on neurotrophin expression and neurogenesis compared to bacterial transplantation alone. These results provide new evidence that gut microbes, their metabolites, or whole fecal content from EE-exposed mice may offer potential therapeutic strategies to address neuropsychiatric disorders.

## 2 Materials and methods

### 2.1 Mice and environmental enrichment protocol

All the experiments conducted were approved by the Italian Ministry of Health (authorization no. 775/2020-PR) under the guidelines of the European Community Council Directive (2010/63/EU) and from Italian D.Lgs 26/2014 for the ethical use of animals in laboratory research. Male C57BL6/N mice, 3 or 4 weeks old, were purchased from Charles River (Calco, Italy). Only male mice were used to avoid possible sex-specific alteration in microbial content (Snigdha et al., 2022). Mice were housed under a 12-h light/dark cycle with animal room’s temperature around 20°C–23°C in standard cages (30 cm × 16 cm × 11 cm) with autoclaved drinking water, nesting material composed by strips of paper (Sizzle-pad, Caipet), and standard chow (Altromin, 1310) *ad libitum*. For the EE protocol, at least ten 3-weeks-old mice were raised in a larger cage (36 cm × 54 cm × 19 cm) with different stimuli: two running wheels, tubes, house and plastic objects for 5 weeks, changing tools twice a week respecting the EE setting with different position of tools (Marrocco et al., 2022). Three or two mice were housed in SE cages. In both experimental groups, the bedding materials were changed once a week.

### 2.2 Stool collection and processing

At the end of the different treatments, fresh stools were collected, frozen, and stored for metagenomic analysis of bacterial composition. Fecal bacterial DNA was extracted with a QIAmp fast DNA Stool mini-kit (51604, QIAGEN) according to the manufacturer’s instructions.

### 2.3 Library preparation and sequencing

Library preparation has been carried out using primer combination Pro341F (CCTACGGGNBGCASCAG) and Pro805R (GACTACNVGGGTATCTAATCC) to amplify the V3-V4 region of 16S rRNA. Subsequently, all samples have been sequenced in 300 paired-end with an Illumina MiSeq platform.

### 2.4 Bioinformatic analysis

Raw fastq files were analyzed with DADA2 pipeline v.1.14 for quality check and filtering (sequencing errors, denoising, chimerae detection). Filtering parameters were as follows: truncLen = 0, minLen = 100, maxN = 0, maxEE = 2, truncQ = 11, trimLeft = 15. All the other parameters in the DADA2 pipeline for paired-end were left as default. Bioinformatic and statistical analyses on recognized ASV were performed with Python v.3.8.2. Each ASV sequence underwent a nucleotide Blast using the National Center for Biotechnology Information (NCBI) Blast software (ncbi-blast-2.3.0) and the latest NCBI 16 S Microbial Database (https://ftp://ftp.ncbi.nlm.nih.gov/blast/db/). Bacterial species present in blanks (washing and water samples) and not having a biological meaning (environmental, rumen, extra-mammals, food, etc.) were excluded, thus the resulting species were considered for subsequent statistical analyses. The relative species abundances used are available in Supplementary Table 1.

### 2.5 Statistics and reproducibility

Measurements of α diversity (within sample diversity) such as Richness and Shannon index, were calculated at species level using the SciKit-learn package v1.0.1, starting from raw reads counts. For beta-diversity, data matrices were first transformed with pseudocount and centered-log-ratio (CLR), then normalized and standardized using QuantileTransformer and StandardScaler methods from Sci-Kit learn package v1.0.1. Normalization using the output_distribution = “normal” option transforms each variable to a Gaussian-like shaped distribution, whilst the standardization results in each normalized variable having a mean of zero and variance of one. Exploratory analysis of β-diversity (between sample diversity) was calculated using the Bray-Curtis measure of dissimilarity and represented in Principal Coordinate Analyses (PcoA), along with methods to compare groups of multivariate sample units (analysis of similarities - ANOSIM, permutational multivariate analysis of variance - PERMANOVA) to assess significance in data points clustering. ANOSIM and PERMANOVA were automatically calculated after 999 permutations, implemented with custom scripts (Python v3.8.2, Seaborn v0.11.2, SciKit-learn v1.0.1). We implemented Partial Least Square Discriminant Analysis (PLS-DA) and the subsequent Variable Importance Plot (VIP) as a supervised analysis wherein the VIP values (order of magnitude) are used to identify the most discriminant bacterial species among the cohorts. Mann–Whitney U test and Kruskal–Wallis test were employed to assess significance for pairwise or multiple comparisons, respectively, considering a P value< =0.05 as significant. Where clearly stated, all P values were corrected for multiple hypothesis testing using a two-stage Benjamini–Hochberg FDR at 10%.

### 2.6 EE-related bacteria transplantation

All the following lyophilized bacteria administered to mice by oral gavage were obtained from Leibniz-institute DSMZ GMBH: *Bacteroides gallinarum* (DSM18171), *Parasutterella excrementishominis* (DSM21040), *Catabacter hongkongensis* (DSM18959), *Alistipes senegalensis* (DSM25460), *Clostridium Kluyveri* (DSM555). These bacteria were selected from those previously identified in mice reared in an enriched environment (Marrocco et al., 2022). The bacteria were resuspended and activated each time before the administration. Briefly, five different vials each containing a strain were broken under the radial sterilizing field of the Bunsen burner apparatus Each lyophilized strain was resuspended in the anaerobe Wilkins Chalgren medium II (1568, Condalab) according to the manufacturer’s procedures (Handling and Safety Information, DSMZ). The amount of bacteria administered via oral gavage was around 6.56 $X\;10^{8}$ per strain, according to the 600 nm absorbance values measured. To prepare the gut to receive the new bacteria cohort, mice were treated with an antibiotic cocktail in the drinking water for 3 days. The bacterial oral gavages started after 1 day of washout from antibiotics and continued for 4 weeks once a week. Each mouse received 200 μL of bacterial cocktail or vehicle (the same amount of anaerobic medium only).

### 2.7 Antibiotic pre-treatment

To maximize the bacterial implantation in the gut, an antibiotic cocktail composed of ampicillin (1 mg/mL, ampicillin ready to use, ThermoFisher, J66972-AB), colistin (1 mg/mL, colistin sulfate, PanReac Applichen, 2922,0001) and streptomycin (5 mg/mL, streptomycin sulfate, PanReac Applichen 1852,0100), as previously described (Terrisse et al., 2021) was given to mice for 3 days before the oral gavage treatment of EE-related bacterial or vehicle. Mice subjected to fecal material transplantation were not pre-treated with antibiotics.

### 2.8 Fecal material transplantation

To prepare the samples for fecal material transplantation (FMT), three pieces of fresh stools from each mouse housed in EE or SE were collected in tubes containing 0.5 mL of PBS. The samples were gently resuspended with an inoculation loop (HS81121C, Merck), and then gently centrifuged (800 g for 3 min) to divide the undigested part from fecal water. To perform FMT we collected 200 μL of fecal water from each animal (donors) and gave the fecal water to receiver mice in a ratio of 1:1.

### 2.9 Behavioral test

Before testing, the mice were placed in the experimental environments for habituation (>10 min). After each test, the apparatus was carefully cleaned with 50% ethanol. The open field task was performed to study general locomotor activity, anxiety behavior, and willingness to explore after bacteria and fecal material transplantation. Mice were placed on the side of the arena (40 × 40 × 30 cm) The total distance travelled, movement duration and time spent in the center of arena (20 cm × 20 cm) were recorded for each mouse, for 10 min with ANY-MAZE software.

### 2.10 Immunofluorescence

Mice were sacrificed and intracardially perfused with PBS and PFA 4%. The brains were isolated and then post-fixed in PFA 4% for 24 h, cryopreserved with 30% sucrose, and frozen. Immunofluorescence staining was performed on cryostat sections (10 µm) of the hippocampal dentate gyrus (DG) region (one section every 240 μm, in the range of −1.46 mm to −2.80 mm from Bregma), the entire DG was acquired using a 20x scan slide. The brain sections were incubated for 1 h with 3% goat serum and 0.3% Triton-X-100, in PBS 0.1 M at RT. Sections were then incubated with rabbit anti-DCX (4604, Cell Signaling, USA) diluted in 1% goat serum and 0.1% Triton-X-100, in PBS 0.1 M at 4°C overnight. After washing in PBS, the sections were incubated with secondary antibody (donkey anti-rabbit, Alexafluor, Invitrogen) for 1 h and Hoechst (33342, Molecular Probes) for 5 min, washed and mounted on a microscope slide for the analysis of fluorescence (Eclipse, Nikon). The number DCX + cells in the DG were counted and normalized to the DG area by MetaMorph software.

### 2.11 RNA extraction and real-time PCR

Total mRNAs were collected from hippocampus of brain sections with RNeasy FFPE kit (73504, QIAGEN) according to the manufacturer’s instructions. The purity and quantity of mRNA was evaluated with Nanodrop One System (Thermo Scientific). One µg of total RNA was reverse transcribed (Mj Mini Thermal Cycler Biorad) using iScriptTM Reverse Transcription Supermix (Biorad) following the manufacturer’s protocol: incubation at 25°C for 5 min, reverse transcription at 46°C for 20 min, inactivation 95°C for 1 min. Real-Time PCR (RT-PCR) was performed in a CFx Real-Time PCR System (Biorad) using SsoFastTM EvaGreen Supermix (Biorad) according to the manufacturer’s instructions. Specific primer pairs, at a final concentration of 500 nM, were used to measure mRNA levels as follows: glyceraldehyde 3-phosphate dehydrogenase (gapdh) F5′-TTCGCAAAACAAGTTCACCA-3′ and R 5′-TCGTTGTGGTTGTA AATGGAA-3′, brain-derived neurotrophic factor (bdnf) F5′-CCATAAG GACGCGGACTTGTAC-3′ and R 5′-AGACATGTTTGCGGCATCCAGG-3′, nerve growth factor (ngf) F: 5′-ACA CTC TGA TCA CTG CGT TTT TG-3′ and R: 5′-CCT TCT GGG ACA TTG CTA TCT GT-3′, epidermal growth factor (Egf) F5′- AGC ATA CTC AGC GTC ACA GC-3′ and R 5′-GCA GGA CCG GCA CAA GTC-3′R′, vascular endothelial growth factor-a (Vegf-α)F5′-GAT CAT GCG GAT CAA ACC TC-3′, and R 5′-AAT GCT TTC TCC GCT CTG AA-3′. The PCR protocol consisted of 40 cycles of denaturation at 95°C for 30 s and annealing/extension at 58°C for 30 s. Melt curve analysis was performed at the end of every RT-q PCR to confirm the formation of a single PCR product. No template controls were added for each target to exclude possible sample contamination. The comparative threshold cycle (Ct) method was used for quantification analysis. The Ct values from each gene were normalized to the Ct value of Gapdh in the same RNA samples. Relative quantification was performed using the 2^−ΔΔCT^ method and expressed as fold changes.

### 2.12 Statistical analysis

The number of replicates (n) for each experiment and the details of statistical analyses are described in the figure legends or main text. Data are presented as mean ± SEM. Statistical analysis were performed using GraphPad Prism 9 software. Exact p-values are given in the text and multiplicity adjusted p-values are given in the corresponding figures (*p < 0.05, **p < 0.01, ***p < 0.001). Unpaired Student’s t-test is used for immunofluorescence analysis, real-time PCR.

## 3 Results

### 3.1 Administration of EE-related bacteria affects anxiety-like behavior and neurotrophins expression in mice

**FIGURE 1:**
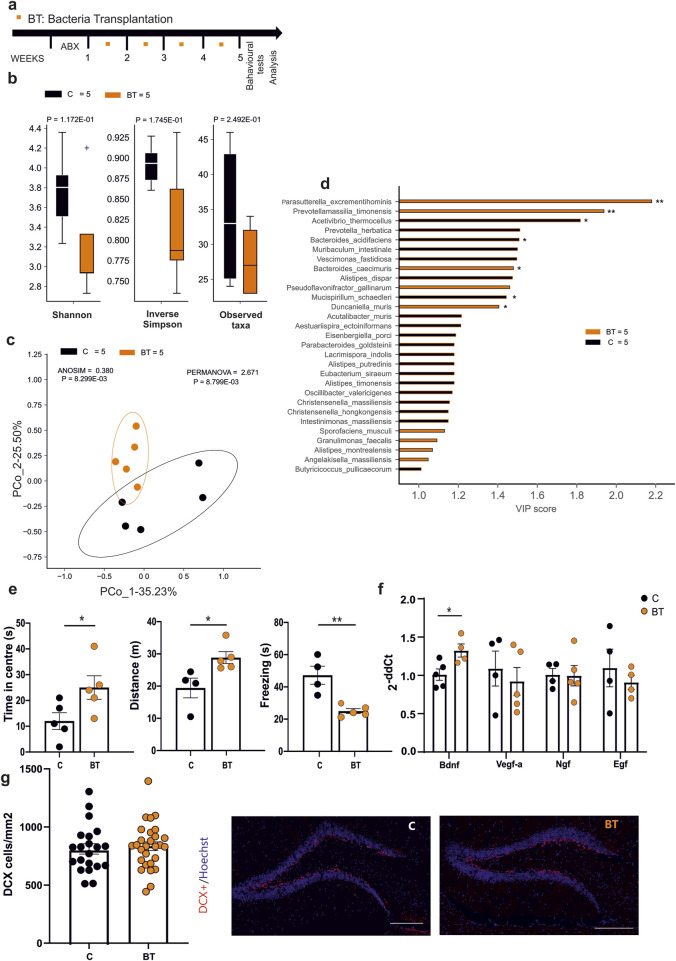
**(a)** Experimental protocol for EE-related Bacteria Transplantation (BT), including antibiotics treatment (3 days) and numbers of oral gavage (orange squares) per week. **(b)** Alpha-diversity (Shannon index, inverse Simpson and observed taxa) showed no differences in both biodiversity and richness metrics after the treatment (C n = 5 and BT n = 5). **(c)** Beta-diversity by the Bray-Curtis dissimilarity distance algorithm showed a significant separation of microbial communities in BT and control mice (C n = 5 and BT n = 5). **(d)** Variable Importance Plot (VIP) showed discriminant species after PLS-DA in descending order of VIP score (bar length), the highest relative abundance depending on the cohort (central bar color) and the lowest one (edge bar color); significant difference after Mann–Whitney U test (non-FDR, *P ≤ 0.05, **P ≤ 0.01; ***P ≤ 0.001) between BT (n = 5) and Control (n = 5) groups **(e)** Open field test showed differences in time spent in the center (n = 5 Control and n = 5 BT) of the arena, distance moved (n = 4 Control and n = 5 BT) and time of the freezing episodes (n = 4 Control and n = 5 BT) (*p = 0.0498; *p = 0.0296; **p = 0.0042, by Student’s t-test, respectively). **(f)** RT-qPCR analysis of hippocampal neurotrophic factors: bar graphs showed increased level of Bdnf (p < 0.05 *, Student’s t-test, two-tailed) in BT compared to control mice. No differences were found in Vegf-a, NGF, and EGF levels. Circles in the bar graph represent the number of samples (1 hippocampus from one animal) used for gene expression analysis. **(g)** Analysis of the neurogenesis in the hippocampal dentate gyrus (DG) in BT or control mice. Circles in the bar graphs show the total number of DCX + cells divided for total area of DG in mm^2^ in each slice analyzed (n = 4 Control animals, 21 slices and n = 5 BT animals, 28 slices; ns p = 0.50, by unpaired t-test). Data are presented as mean ± s.e.m. (g right): representative images of mouse hippocampal DG coronal sections (scan slides at ×20 magnification): DCX + cells (red) and nuclei (Hoechst, blue) in control and BT mice (scale bar, 100 μm).

We previously demonstrated that housing mice in an EE affects their microbiota and metabolome composition, modulating hippocampal plasticity and gene expression (Marrocco et al., 2022). In particular, we identified a number of bacterial species characterizing the EE housing conditions and correlating with EE-induced changes (Marrocco et al., 2022). Now, we investigated whether treating mice with EE-related bacterial cohorts could affect mice behavior and brain plasticity. At this aim we treated mice with a bacterial consortium composed by *B. gallinarum, Parasutterella excrementihominis, Catabacter hongkongensis (*recently named *Christensenella hongkongensis), A. senegalensis, Clostridium kluyveri,* by oral gavage, once a week for four times. Three days pre-treatment with an antibiotic cocktail was performed to increase colonization of transplanted bacteria (Figure 1a). The fecal microbiota of mice undergoing bacterial transplantation (BT) showed no differences in species diversity (Shannon and inverse Simpson index, Figure 1b) or richness (Observed taxa, BT [n = 5]; C [n = 5] by Mann-Whitney test), when compared to the control group. At difference, the Bray-Curtis dissimilarity distance, employed to quantify beta diversity, showed a significant separation between BT and control groups suggesting that EE-related bacteria induced changes in terms of the composition of gut bacteria community (Figure 1c). Interestingly, the beta diversity metrics are affected by EE-related bacteria administration similarly to what previously reported for EE-housed mice (Marrocco et al., 2022). In addition, we performed Partial Least Square Discriminant Analysis (PLS-DA) and Variable Importance Plot (VIP) analysis to evaluate the colonization of the transplanted species. As shown in Figure 1d among the species highly associated with BT mice, we found that *P. excrementihominis* was the most representative species while none of the other transplanted species changed significantly compared to controls. To evaluate the effects of EE-related BT on anxiety-like behavior in mice, 10 min open field tests were performed. Three days after the last gavage, BT mice showed significant increases of the time spent in the center of the arena (Figure 1e, C [n = 5], 12 ± 3 s; BT [n = 5], 25 ± 4 s, *p = 0.0498, Student’s t-test) and of the total distance moved (C [n = 4], 19.41 ± 3.06 m; BT [n = 5] 28.6 ± 1.8 m, *p = 0.0296, Student’s t-test). In accordance, reduced times of immobility (freezing) were observed in BT mice compared to controls (C [n = 4], 47.2 ± 5.6 s; BT [n = 5], 25.4 ± 1.5 s, **p = 0.0042, Student’s t-test). We also verified that observed changes in behavior were not due to the administration of the antibiotics as shown in Supplementary Figure S1. To further investigate whether BT could modulate hippocampal gene expression, as reported for EE housed mice, quantitative PCR analysis was performed. Data reported in Figure 1f demonstrate an increased level of Bdnf (C [n = 5], 1.01 ± 0.07; BT [n = 4], 1.30 ± 0.08 *p < 0.0272; Student’s t-test), while other neurotrophins were not modified by BT. Similarly, no difference in the number of DCX + positive cells in the dentate gyrus, indicative of new neuronal precursors generation (Figure 1g) was observed upon BT. These data indicate that the administration of EE-related bacteria partially replicates the beneficial effects EE on the brain.

### 3.2 Transplantation of fecal material from mice housed in EE affects anxiety-like behavior, neurotrophins expression and neurogenesis in receiving mice

**FIGURE 2:**
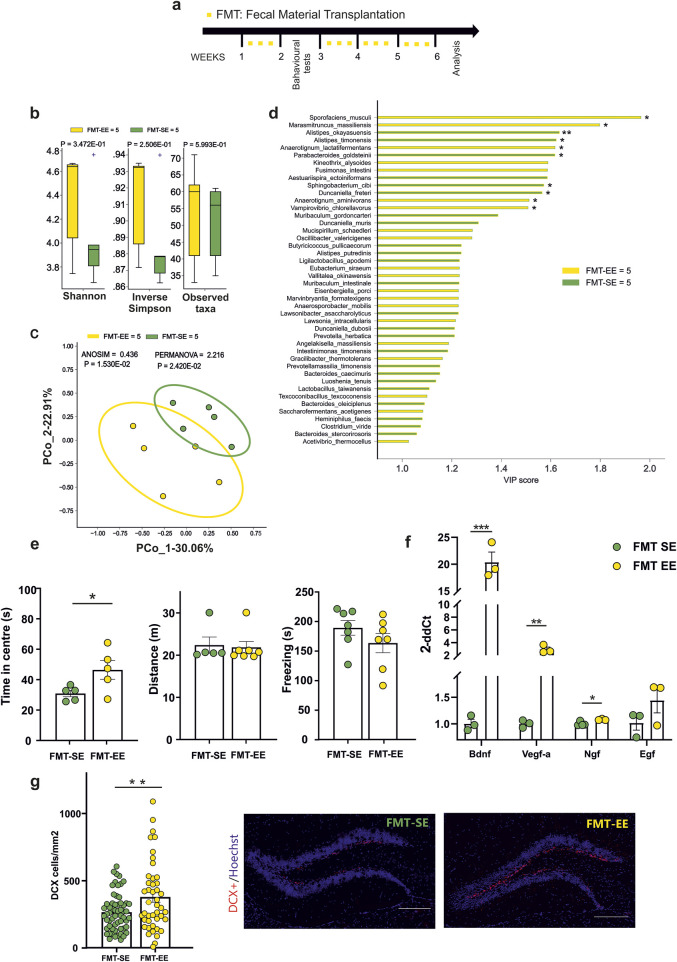
**(a)** Experimental protocol for Fecal Material Transplantation (FMT): oral gavage of fecal content derived from mice reared in an enriched environment (EE) or standard environment (SE) were performed three times a week for four times (yellow squares). The behavioural test were performed after 1 week from treatment **(b)** Alpha- (Shannon, inverse Simpson) and Beta-diversity showed no differences in FMT-SE (n = 5) vs. FMT-EE (n = 5). **(c)** Bray-Curtis distance dissimilarity algorithm showed a significant separation of the microbial cohorts between FMT-SE and FMT-EE groups. **(d)** Variable Importance Plot (VIP) showed discriminant species after PLS-DA in descending order of VIP score (bar length), the highest relative abundance depending on the cohort (central bar color) and the lowest one (edge bar color); significant difference after Mann–Whitney U test (non-FDR, *P ≤ 0.05, **P ≤ 0.01; ***P ≤ 0.001) between FMT-SE (n = 5) and FMT-EE (n = 5) groups **(e)** Behavioral tests showed differences in the time spent in the center of the arena for FMT-EE (n = 5) compared to FMT-SE mice (n = 5 *p = 0,0473, Student’s t-test). **(f)** RT-qPCR analysis of hippocampal neurotrophic factors: an increased level of Bdnf, Vegf-a and Ngf (***p = 0.0005, **p = 0.006, *p < 0.02, Student’s t-test, two-tailed) in FMT-EE (n = 3) compared to FMT-SE mice (n = 3–4) was observed. No differences were found in EGF levels between groups. **(g)** The neurogenesis analysis in the dentate gyrus (DG) after FMT from EE or SE mice. Circles in the bar graphs show the total number of DCX + cells divided for total area of DG in mm^2^ in each slice analyzed (n = 6 FMT-SE, 59 slices and n = 3 FMT-EE animals, 44 slices; **p = 0.0051, by unpaired t-test). Data are presented as mean ± s.e.m (g right). Representative images of hippocampal DG coronal sections stained for DCX + cells (red) and nuclei (Hoechst, blue) in FMT-Se and FMT-EE mice (scale bar, 100 μm). All data are expressed as mean ± s.e.m.

As alternative approach to transfer the beneficial effects of EE on brain plasticity and anxiety-like behavior, we investigated the effects of fecal material transplantation from EE housed mice to control SE-housed mice. At this aim, stools were collected from donor mice living either in EE or SE (for at least 4 weeks), transplanted to receiving mice housed in SE by oral gavage (three times a week) and tested as shown in Figure 2a. Donors are thereafter indicated as SE- or EE-mice and receivers as FMT-SE- or FMT-EE-mice. We first assessed whether donor mice housed in EE showed less anxiety-like behaviour compared to SE donors, and at the end of the experiments we also examined the microbiota composition of both groups. As shown in Supplementary Figure S2, EE donor mice showed less anxiety-like behaviour as well as differences in alpha and beta diversity as previously shown (Marrocco et al., 2022), while PLS-DA VIP analysis partially recapitulated enriched species previously found in EE and SE. At the end of treatments, no significant differences were observed in biodiversity or richness of the fecal microbiota among FMT-SE and FMT-EE mice Figure 2b by Mann-Whitney test. Nevertheless, the Bray-Curtis dissimilarity distance algorithm showed a clear separation (Figure 2c). These data suggest that FMT transfer from mice housed in different environments mainly affects the interactions among microbial communities in the receiving mice. To possibly identify bacterial species distinctive for FMT-EE or FMT-SE cohorts, PLS-DA model and the VIP score were implemented. At least twenty species were distinctive for the EE group, and seven were more significantly enriched in EE, as shown in the pairwise analysis (Figure 2d). Among them *Sporofaciens musculi, Marasmitruncus massiliensis* showed the highest VIP scores possibly being involved in the effects observed. To further evaluate the effect of the different FMT treatments on receiver behavior, the open field tests were performed. The results of the test showed an increased time spent in the center of the arena for the FMT-EE compared to FMT-SE mice (Figure 2e, FMT SE = [n = 5], 30.88 ± 2.07 s; FMT EE = [n = 5], 46.24 ± 6.22 s, *p = 0.0473; Student’s t-test). No differences in the distance travelled and in freezing behavior were observed between the two groups. We also analyzed the effects of FMT treatment on the expression of neurotrophic factors and on neurogenesis in the hippocampal region. The analysis of gene expression showed significant changes in Bdnf (FMT-SE [n = 3] 1.006 ± 0.080; FMT-EE [n = 3] 20.390 ± 1.874, ***p = 0.0005), Vegf (FMT-SE [n = 3],1.002 ± 0.040; FMT-EE n = 3, 2.929 ± 0.369, **p = 0.006) and Ngf (FMT-SE, [n = 4]; 1.001 ± 0.019; FMT-EE [n = 3],1.081 ± 0.007, *p = 0.02 by unpaired t-test) (Figure 2f). In addition, we observed a significant increase of DCX + positive cells in FMT-EE mice compared to FMT-SE, emphasizing the role of gut contents on brain function (FMT SE [n = 59], 266.4 ± 18 cells/mm^2^; FMT EE [n = 44], mean 379.1 ± 38.6 cells/mm^2^, **p = 0.0051; Student’s t-test) (Figure 2g). Altogether these data demonstrate that several functions modulated by environmental housing conditions in mice could be transferred to SE housed mice through the fecal content of EE-housed mice.

## 4 Discussion

It has been described that gut microbiota composition participates in the regulation of host homeostasis and several recent evidence suggest direct and indirect implications of microbes in the modulation of brain microenvironment and functions (Rogers et al., 2016; Sorboni et al., 2022). The use of probiotics reduces the behavioral deficits and restores the microbiota composition in patients with major depressive disorder (MDD) (Huang et al., 2016) alleviating the gastrointestinal and psychiatric symptoms (Johnson et al., 2023). Microbe transplantation from healthy donors reduces memory loss in Alzheimer patients and mice models of the disease (Nassar et al., 2022), while treating mice with microbes identified in AD patients induces endoplasmic reticulum stress (Liu et al., 2023) suggesting a possible role of microbes in disease etiology.

In this work we investigated the bi-directional communication between the gut and the brain in physiologic conditions, focusing our attention on the effect of a healthy lifestyle, modeled by housing mice in an EE. In previous work (Marrocco et al., 2022) we described the specific microbial profile associated with EE housed mice, composed of five bacteria such as *B. gallinarum, P. excrementihominis, Catabacter hongkongensis, A. senegalensis, C. kluyveri*. In this study we further investigated the hypothesis of the involvement of gut microbiota in EE-induced changes in the brain and we observed behavioral, molecular and metagenomic changes upon transfer of bacteria or fecal material. We identify different microbial populations in the two experimental conditions and we wonder whether the oral administration of specific bacteria or fresh fecal material could modify the microbial community in the receivers. We demonstrated that changing the microbiota composition by transferring fecal material or a defined bacterial consortium improved the behavioral outcomes in mice, reducing anxiety-like behavior. The efficacy of fecal material was stronger in comparison with BT, likely suggesting that the two treatments act differently. We cannot exclude differences due to the timing of animal manipulation or to different microbes proliferation in the receivers due to different nature of the gavage content (lyophilized or fresh stool). Studying the effects of BT and FMT on the expression of neurotrophic genes in the hippocampal region, we observed an increase in BDNF gene expression levels in both groups. BDNF represents one of the most common biomarkers (Thornton, 2023) of a healthy brain and lower serum BDNF levels are associated with a higher risk of depressive symptoms in women with cardiovascular disease (Medved et al., 2024). In FMT-treated mice, additional neurotrophins are modulated, with increase of Vegf-a and NGF and higher neurogenesis in the dentate gyrus. The effects on gene expression observed in the FMT group are in line with previous results showing that the administration of SCFAs (formate and acetate) induced changes similar to EE (Marrocco et al., 2022). Moreover, the analysis of fecal bacterial content in BT mice revealed a significant presence of *P. excrementihominis* among all the bacteria administered via oral gavage. This microbe was strongly correlated with formate levels, one of the short-chain fatty acids that play an important role in EE-like changes (Marrocco et al., 2022). However, our data show that other different species were specifically enriched in BT mice after ABX pre-treatment plus inoculation of the bacterial cohort compared to mice receiving only ABX pre-treatment, suggesting that the globally different microbiome (and possibly the relative metabolome) obtained by specific cohort inoculation may be responsible for the differences between the two groups (C vs BT).

The analysis of microbial profile in the EE donor mice partially overlapped the one seen in our previous work; however, the behavioural outcomes of EE group were in line with the literature in terms of anxiety-like behavior, and the microbial profile identified was segregated from SE donor group, highlighting the validation of these mice as donors for the FMT experiments. Note that the alpha diversity of mice receiving fecal material from SE or EE housed mice does not replicate the effects induced by the housing conditions (Marrocco et al., 2022), likely suggesting that FMT-induced effects on host microbiota could hide more subtle effects evoked by transplantation of fecal content from mice housed in different conditions.

The fecal bacteria analysis of FMT partially replicated the segregation of microbial profile observed in the donors; however, we identified some new bacterial strains, such as *Sporofaciens musculi and Anaerotignum lactifermentas* in both EE donors and FMT EE mice. These Gram-positive microbes were isolated for the first time from the caecum of mice and broiler chicken respectively (Rasmussen et al., 2019; Van Der Wielen et al., 2002) and their main metabolic product is acetate. In FMT-EE mice, *Kineothrix alysoides* produces as main metabolites acetate and formate (Haas and Blanchard, 2017) while *Anaerotignum aminovorans* produces acetate as main metabolite (Ueki et al., 2017). Of note, acetate and formate are the metabolites strongly associated with EE and were responsible of the beneficial effects on the central nervous system as observed in our previous work.

In conclusion, we propose that housing mice in an enriched environment leads to profound alterations in the composition of both the microbiota and metabolome. These findings not only highlight the intricate interplay between environmental stimuli and neurobiological processes, but also raise compelling questions about the extent to which external conditions can shape brain function through the gut-brain axis. This insight paves the way for a deeper exploration of how lifestyle and environment may serve as powerful modulators of mental health, potentially offering novel avenues for therapeutic interventions in neurological and psychiatric disorders.

## 5 Limitations of the study

Considering the differences induced by FMT and BT treatments, we cannot exclude that metabolites present in the fecal water could play additional roles, according to our previous work (Marrocco et al., 2022), and highlighting the presence of different microbial profiles showing the same behavioural outcome in EE mice. In addition, we must consider that oral gavage represents a potentially stressful procedure (Walker et al., 2012) and that this modality of bacteria administration can modify anxiety-like behavior in mice. Further work will be necessary to elucidate the mechanism underlying the effect of EE-induced microbiota on the brain, but here we suggest that enriched environment induces changes in the composition of the microbiota to possibly create different ecological structures (bacteria and metabolome) compared to SE-housed mice, involved in the beneficial effects of the enriched environment in mice.

## Data Availability

The datasets generated and analyzed for this study can be found in the Figshare repository (10.6084/m9.figshare.28369859).
