# Supplementary material for: Fecal or bacterial transplantation in mice transfer environment-induced brain plasticity and associated behavioral changes
Source: Front Physiol. 2025 May 30;16:1572854. doi: 10.3389/fphys.2025.1572854 (PMC12162564; doi:10.3389/fphys.2025.1572854)
Supplement: Supplementary file 1 [file DataSheet1.pdf]

Supplementary Material

Supplementary Figures

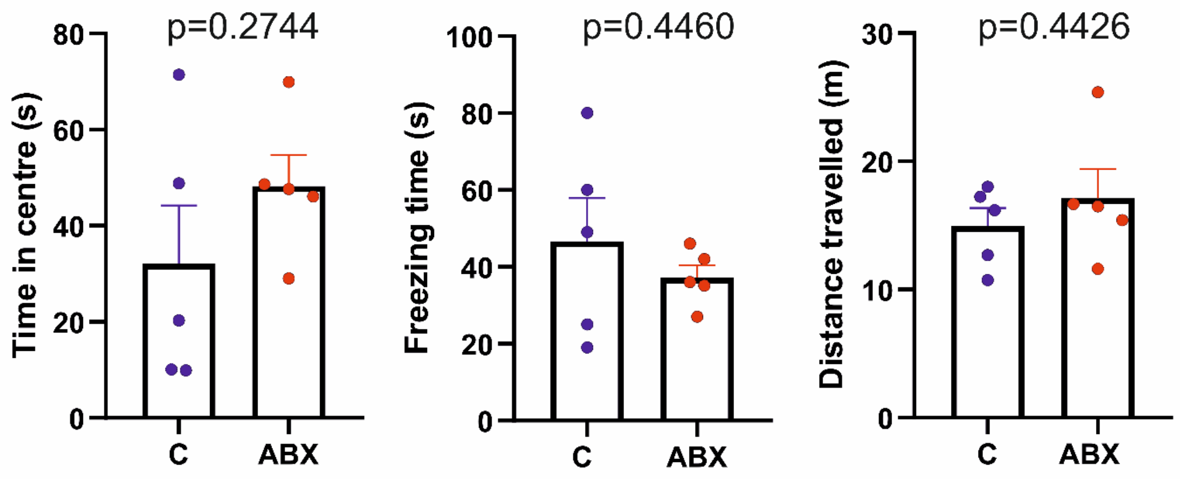

Supplementary Figure 1. Open field test for control (C, n=5) and ABX-pretreated (ABX, n=5) mice. ABX mice were treated with or without antibiotic cocktail in the drinking water for three days and anxiety-like behaviour was tested after a further four weeks. Both groups received weekly PBS treatment by oral gavage as shown in Figure 1a. None of the parameters analysed showed a significant difference between C and ABX mice by unpaired Student's t-test.

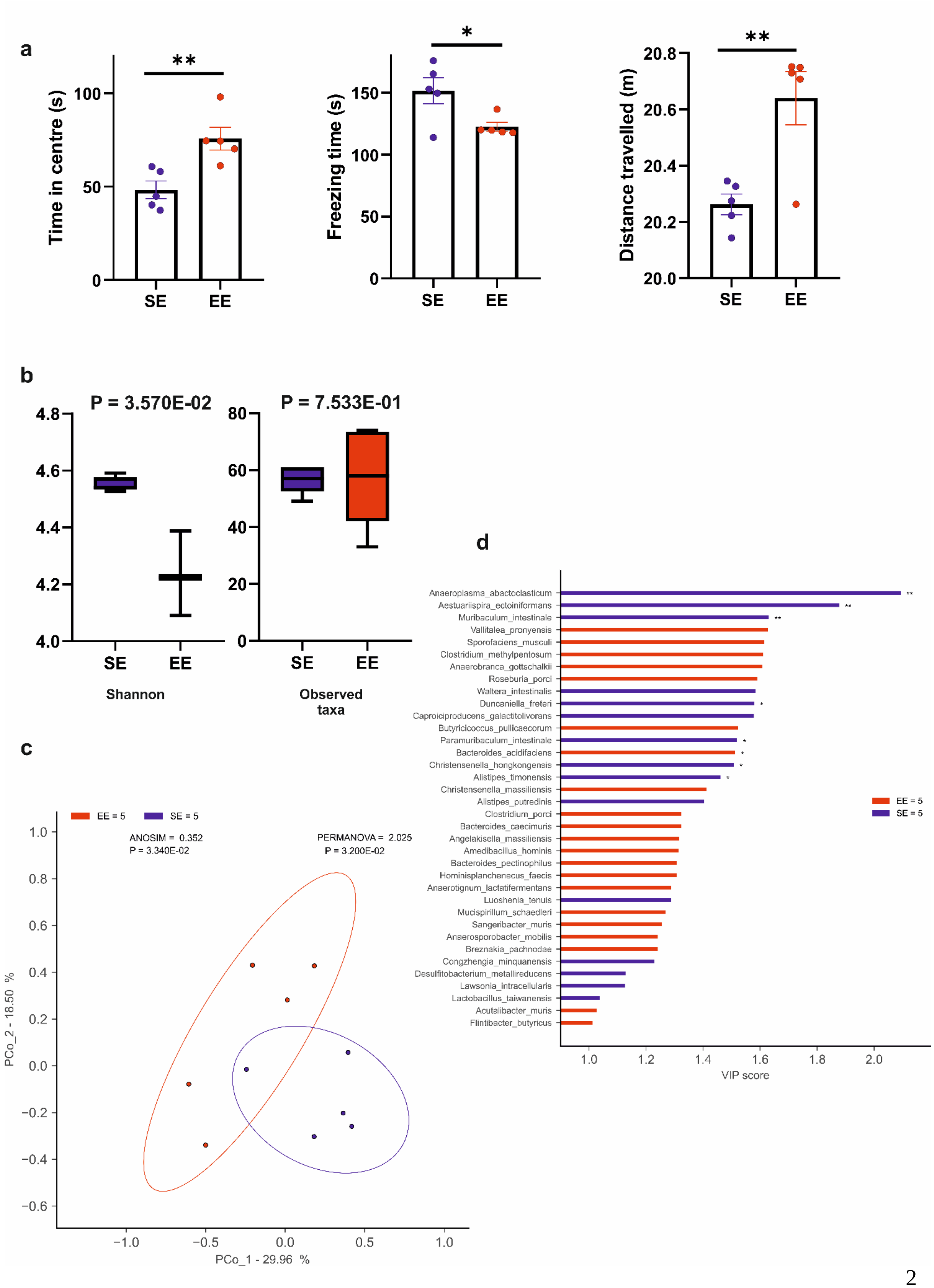
a
Time in centre (s)
Freezing time (s)
Distance travelled (m)
SE
EE
**
*
b
P = 3.570E-02
P = 7.533E-01
Shannon
Observed taxa
c
EE = 5
SE = 5
ANOSIM = 0.352
P = 3.340E-02
PERMANOVA = 2.025
P = 3.200E-02
PCo_1 - 29.96 %
PCo_2 - 18.50 %
d
Anaeroplasma_abactoclasticum
Aestuariispira_ectoiniformans
Muribaculum_intestinale
Vallitalea_pronyensis
Sporofaciens_musculi
Clostridium_methylpentosum
Anaerobranca_gottschalkii
Roseburia_porci
Waltera_intestinalis
Duncaniella_freteri
Caproiciproducens_galactitolivorans
Butyricicoccus_pullicaecorum
Paramuribaculum_intestinale
Bacteroides_acidifaciens
Christensenella_hongkongensis
Alistipes_timonensis
Christensenella_massiliensis
Alistipes_putredinis
Clostridium_porci
Bacteroides_caecimuris
Angelakisella_massiliensis
Amedibacillus_hominis
Bacteroides_pectinophilus
Hominisplanchenecus_faecis
Anaerotignum_lactatifermentans
Luoshenia_tenuis
Mucispirillum_schaedleri
Sangeribacter_muris
Anaerosporobacter_mobilis
Breznakia_pachnodae
Congzhengia_minquanensis
Desulfitobacterium_metallireducens
Lawsonia_intracellularis
Lactobacillus_taiwanensis
Acutalibacter_muris
Flintibacter_butyricus
VIP score
EE = 5
SE = 5

Supplementary Figure 2. a: open field test for standard (SE, n=5) and enriched (EE, n=5) donor mice. EE mice spent more time in the centre of the arena (EE= 75.73±6.09, SE=48.28±4.73, **p=0.0075), experienced less freezing time (EE= 122.5±3.5, SE=151.5±10.4, *p=0.0306) and travelled more (EE= 20.64±0.09, SE=20.26±0.03, **p=0.0059 by unpaired Student's t-test) than SE mice. b: Alpha diversity analysis of bacterial DNA isolated from stool of SE and EE mice at the end of experiment. EE mice showed significant decrease in species biodiversity (Shannon index) while richness (number of taxa) was not different compared to SE mice by Mann–Whitney *U* test. C: beta- diversity analysis of bacterial community in SE and EE mice. Bray-Curtis dissimilarity distance showed a significant separation between donor Se and EE mice. d: Variable Importance Plot (VIP) showed discriminant species after PLS-DA in descending order of VIP score (bar length), the highest relative abundance depending on the cohort (central bar color) and the lowest one (edge bar color); significant difference after Mann–Whitney *U* test (non-FDR, $*P \leq 0.05$, $**P \leq 0.01$; $***P \leq 0.001$) in the two donor mice groups (SE and EE).

.
